# Allergic diseases in children and adolescents in Germany. Results of the cross-sectional KiGGS Wave 2 study and trends

**DOI:** 10.17886/RKI-GBE-2018-082

**Published:** 2018-09-19

**Authors:** Roma Thamm, Christina Poethko-Müller, Antje Hüther, Michael Thamm

**Affiliations:** Robert Koch Institute, Berlin, Department of Epidemiology and Health Monitoring

**Keywords:** ALLERGIC DISEASES, ALLERGIC SENSITISATION, CHILDREN, ADOLESCENTS, HEALTH MONITORING

## Abstract

Allergic diseases are among the most common health issues children and adolescents face. Allergic reactions occur when the immune system becomes allergically sensitised and are detected by measuring levels of specific immunoglobulin E antibodies (IgE antibodies) in the blood. This article discusses the prevalences of bronchial asthma, hay fever, atopic dermatitis and allergic contact dermatitis for 0- to 17-year-olds, as well as the prevalence of allergic sensitisation to a mix of frequent inhalant allergens (SX1) among 3- to 17-year-olds based on data from the second wave of the German Health Interview and Examination Survey for Children and Adolescents (KiGGS Wave 2, 2014-2017). 12-month prevalence trends between KiGGS Wave 2 and the KiGGS baseline study (2003-2006) are shown according to gender and age group. There were no significant changes in the 12-month prevalence of hay fever (8.8%) atopic dermatitis (7.0%) and bronchial asthma (3.5%) compared to the KiGGS baseline study, which indicates a stabilisation at a high level. More than one in six children (16.1%) currently suffer from at least one of these three diseases. 37.1% of 3- to 17-year-olds are sensitised to the multiple allergen mix SX1. Similar to the development of disease prevalence, SX1 sensitisation too has remained stable at a high level over the course of the past ten years.

## 1. Introduction

Allergic diseases such as hay fever (allergic rhinitis), bronchial asthma and atopic dermatitis (atopic eczema) are among the most common diseases affecting the health of children and adolescents. These diseases often severely impact the daily lives of those affected. Hay fever is an allergic inflammation reaction of the nasal mucosa that causes itching, sneezing fits, increased mucus secretion and obstructed nasal breathing. In many cases the eyes are affected too. A diverse set of allergens such as pollen, moulds, animal epithelial tissue or house dust mites can trigger these complaints. Bronchial asthma is triggered by an oversensitivity of the bronchia to different irritants. This leads to reversible paroxysmal constrictions of the bronchial system and may lead to coughing, wheezing when breathing and dyspnoea. In the majority of children concerned, allergies are the cause of asthma. Atopic dermatitis and allergic contact dermatitis are different diseases of the skin caused by allergy with symptoms such as strong itching, reddening and blistering [1, 2].

**KiGGS Wave 2**

Second follow-up to the German Health Interview and Examination Survey for Children and Adolescents

**Data owner:** Robert Koch Institute

**Aim:** Providing reliable information on health status, health-related behaviour, living conditions, protective and risk factors, and health care among children, adolescents and young adults living in Germany, with the possibility of trend and longitudinal analyses

**Study design:** Combined cross-sectional and cohort study

**Cross-sectional study in KiGGS Wave 2**

**Age range:** 0-17 years

**Population:** Children and adolescents with permanent residence in Germany

**Sampling:** Samples from official residency registries - randomly selected children and adolescents from the 167 cities and municipalities covered by the KiGGS baseline study

**Sample size:** 15,023 participants

**KiGGS cohort study in KiGGS Wave 2**

**Age range:** 10-31 years

**Sampling:** Re-invitation of everyone who took part in the KiGGS baseline study and who was willing to participate in a follow-up

**Sample size:** 10,853 participants

**KiGGS survey waves**

► KiGGS baseline study (2003-2006), examination and interview survey
► KiGGS Wave 1 (2009-2012), interview survey
► KiGGS Wave 2 (2014-2017), examination and interview survey

More information is available at www.kiggs-studie.de/english

Allergic sensitisation of the immune system is a prerequisite of allergies. Asthma, hay fever and atopic dermatitis are caused by specific immunoglobulin E antibodies (IgE antibodies) after an (initial) contact with specific, generally harmless substances (allergens), this is called atopy. With repeated contact with these same allergens, the sensitised immune system remembers these allergens and may react with defence mechanisms. This can lead to allergic reactions in diverse organs with different degrees of severity and symptoms. Allergic sensitisation can be measured by analysing specific IgE antibodies in the blood. Their detection alone, however, is no proof of a disease, but does indicate an increased risk for allergic diseases [3]. For asthma and hay fever, respiratory sensitisers are of particular importance.

In Western industrialised nations, the prevalence of allergic and, in particular, atopic diseases has increased significantly during the second half of the 20th century. The results of the ISAAC study (International Study of Asthma and Allergies in Childhood) and the repeated surveys of school-entry-age children in East and West Germany during the 1990s revealed further, yet fewer, pronounced increases in Germany [4-6]. The prevalence established by the baseline study of the German Health Interview and Examination Survey for Children and Adolescents (KiGGS, 2003-2006) can now be compared with those found in the second wave (2014-2017).

The article presents the lifetime and 12-month prevalences (‘ever’ and ‘during the past twelve months’) for bronchial asthma, hay fever and atopic dermatitis, as well as allergic contact dermatitis for children and adolescents aged 0 to 17. For the 3 to 17 age group, the point prevalence of allergic sensitisation (at the point of blood draw) to a mixture of a respiratory sensitisers (SX1). Moreover, differences (trends) in the 12-month prevalence of atopic diseases, as well as SX1 sensitisation between KiGGS Wave 2 and the KiGGS baseline study are presented according to gender and age group.

## 2. Methodology

KiGGS is part of the health monitoring system at the Robert Koch Institute (RKI) and includes repeated cross-sectional surveys of children and adolescents aged 0 to 17 (KiGGS cross-sectional study) that are representative for Germany. The KiGGS baseline study (2003-2006) was conducted as an examination and interview survey, the first follow-up study (KiGGS Wave 1, 2009-2012) as a telephone-based interview survey and KiGGS Wave 2 (2014-2017) as an examination and interview survey. Previous articles have provided a detailed description of the concept and design of KiGGS [7-10]. In brief, participants to be invited were selected randomly from the official population registries in 167 cities and municipalities representative for Germany and already used in the baseline study. A number of activities have been conducted to increase study participation and to improve the sample composition [8, 11]. 15,023 children and adolescents (7,538 girls, 7,485 boys) took part in KiGGS Wave 2 (response 40.1%). 3,567 children and adolescents (1,801 girls, 1,766 boys) took part in the examinations (response 41.5%).

KiGGS Wave 2 collected data on allergic diseases by using questionnaires which participants were asked to fill in (interview survey participants) or which were surveyed in medical interviews (participants in both the interview and examination). Bronchial asthma, hay fever (allergic rhinitis or allergic conjunctivitis, atopic dermatitis (atopic eczema/endogenous eczema) and allergic contact dermatitis were surveyed through the questions “Has a physician ever diagnosed your child with the <disease X>?”, “Has the disease occurred during the past 12 months?” For children suffering from asthma, hay fever and/or atopic dermatitis parents and/or guardians were additionally asked; “Has your child taken medication for the <disease X> during the last 12 months?” The data collected was used to calculate the lifetime and 12-month prevalence of allergic diseases.

The KiGGS baseline study surveyed data on allergic contact dermatitis for all participants by using self-administered questionnaires. The question was “Did your child ever develop an allergic contact dermatitis (for example a skin rash from nickel in a watch or jewellery)?” Unlike in KiGGS Wave 2, answering ‘yes’ did not depend on having a medical diagnosis in the KiGGS baseline study.

Next to age and gender, the surveyed sociodemographic variables included family socioeconomic status (SES). In KiGGS Wave 2 the socioeconomic status was measured through an index based on the information parents provided on educational background, occupational status and income situation (equivalised disposable income). SES allowed for a differentiation between low, medium and high SES groups [12].

A serum sample was taken from 3- to 17-year-old children and adolescents that participated in KiGGS Wave 2 examinations, and analysed for specific IgE antibodies. The PHADIA (now Thermo Fisher Scientific) IMMUNOCAP testing system was applied for quantitative detection, using the UNICAP 1000 analyser. A value of ≥ 0,35 kU_A_/l was interpreted as a positive test result.

The results were stratified according to gender, age, region of residence (East/West Germany) and SES based on prevalences with 95% confidence intervals (95%-CI). To produce representative findings on regional structure and age (in years), gender, federal state (official population statistics as of 31 December 2015), German citizenship (as of 31 December 2014) as well as parental levels of education according to the Comparative Analysis of Social Mobility in Industrial Nations (CASMIN) classification [13] (micro census 2013 [14]) a corresponding weighting factor was developed for analyses. A separate weighting factor was created for laboratory examinations.

The basis for the description of trends between the KiGGS baseline study (data set version 23) and KiGGS Wave 2 were the age and gender standardised prevalences (as of 31 December 2015) at the point of data collection. New weighting factors were applied to the data from the KiGGS baseline study that were calculated in line with those applied to KiGGS Wave 2 data.

All analyses were conducted with SAS 9.4 (SAS Institute, Cary, North Carolina, USA) based on the KiGGS Wave 2 data set (Version 7). To suitably account for the clustering of participants at sample points and the weighting in the calculation of confidence intervals and p-values, SAS survey procedures were applied in all analyses. A statistically significant difference between groups is assumed to have been demonstrated in cases where the corresponding 95% confidence intervals do not overlap and the p-value was lower than 0.01.

## 3. Results

###  

#### Bronchial asthma

The prevalence of children and adolescents from 0 to 17 years ever receiving a medical diagnosis of asthma in Germany was 6.0%. 3.5% of children and adolescents in this age range also currently suffer from asthma, and that means it occurred during the last 12 months. Asthma was detected far more frequently at least once in a lifetime among boys compared to girls (7.5% vs. 4.5%). Also boys currently suffer from asthma more often than girls (4.4% vs. 2.6%).

The prevalence of asthma increases among girls and boys up to about school age, although the increase is more marked for boys. The SES of parents too influences child asthma prevalences. The lowest prevalence of asthma is found among children and adolescents from families with high SES. A comparison of asthma prevalences between Eastern and Western Germany did not reveal any differences (Table 1 and Table 2).

**Figure 1:**
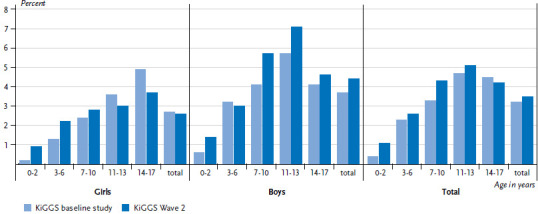
Trends in the 12-month prevalence of asthma between the KiGGS baseline study and KiGGS Wave 2 according to gender and age (KiGGS baseline study n=8,543 girls, n=8,849 boys; KiGGS Wave 2 n=7,402 girls, n=7,317 boys) Source: KiGGS baseline study (2003-2006), KiGGS Wave 2 (2014-2017)

Overall, the 12-month prevalence of asthma has not changed significantly compared to the KiGGS baseline study (KiGGS Wave 2 3.5%, KiGGS baseline study 3.2%). Categorised by gender, prevalence has remained constant for girls (2.6% vs. 2.7%) and slightly increased for boys (4.4% vs. 3.7%). Principally, the increase is owed to the higher prevalence among boys in the age group 7 to 10 years (5.7% vs. 4.1%) and 11 to 13 years (7.1% vs. 5.7%; Figure 1). In absolute figures, nearly half a million children and adolescents in Germany currently suffer from asthma.

#### Hay fever

11.0% of children and adolescents in Germany have been diagnosed with hay fever at least once in their lives. Hay fever has also occurred over the last 12 months among 8.8% of them, i.e. three quarters of these children and adolescents. Compared to girls, boys received a medical diagnosis of hay fever significantly more often (13.0% vs. 8.9%), and boys are also currently affected more frequently (10.4% vs. 7.2%).

The prevalence of hay fever increases continuously among girls and boys with age. The SES of parents has no significant impact on the prevalence of hay fever, either among girls or boys. Moreover, there are no significant differences between Eastern and Western Germany (Table 1 and Table 2).

**Figure 2:**
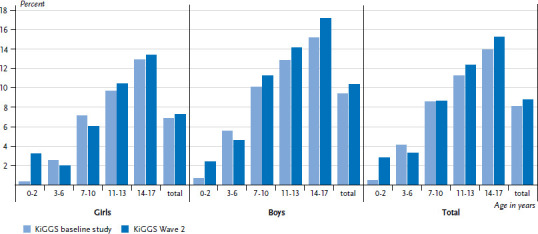
Trends in the 12-month prevalence of hay fever between the KiGGS baseline study and KiGGS Wave 2 according to gender and age (KiGGS baseline study n=8,519 girls, n=8,829 boys; KiGGS Wave 2 n=7,431 girls, n=7,367 boys) Source: KiGGS baseline study (2003-2006), KiGGS Wave 2 (2014-2017)

At the point of the KiGGS baseline study, the 12-month prevalence of hay fever among 0- to 17-year-olds was 8.1%. The current figures confirm that the prevalence of hay fever remains at a constantly high level. The characteristic differences according to gender and age that were observed originally with a higher prevalence among boys compared to girls and a clear increase of prevalence with age in both genders has also remained unchanged (Figure 2). In Germany, therefore more than one million children and adolescents remain affected by hay fever.

#### Atopic dermatitis

With a prevalence of 12.8% children and adolescents have ever been medically diagnosed with atopic dermatitis more frequently compared to hay fever and asthma. According to responses from parents, 7.0% of girls and boys also currently suffer from atopic dermatitis. Children are most often diagnosed with atopic dermatitis at the age of 0 to 2 years. Lifetime prevalences are therefore stable over age groups. The levels of current atopic dermatitis, however, decrease with age and more significantly in boys than girls.

The highest lifetime prevalences for atopic dermatitis are found among children and adolescents from families from the highest SES groups. Categorised according to gender and taking current cases into consideration, the influence of SES is, however, not statistically significant. A clearer correlation with the prevalence of atopic dermatitis can be observed depending on whether a person lives in Eastern or Western Germany. Lifetime prevalence and the number of current cases are higher in Eastern Germany compared to West Germany (Table 1 and Table 2).

**Figure 3:**
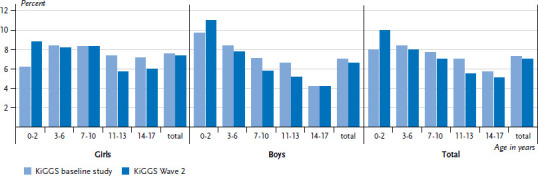
Trends in the 12-month prevalence of atopic dermatitis between the KiGGS baseline study and KiGGS Wave 2 according to gender and age (KiGGS baseline study n=8,482 girls, n=8,787 boys; KiGGS Wave 2 n=7,381 girls, n=7,341 boys) Source: KiGGS baseline study (2003-2006), KiGGS Wave 2 (2014-2017)

Compared to the KiGGS baseline study, no change in the number of current cases of atopic dermatitis has been observed (KiGGS Wave 2 7.0%, KiGGS baseline study 7.3%). Moreover, girls continue to be affected more often than boys (Figure 3). Expressed in absolute figures, around 900,000 children and adolescents in Germany suffer from atopic dermatitis.

#### At least one atopic disease

Grouping the three atopic diseases asthma, hay fever and atopic dermatitis together as ‘atopic diseases’, 23.7% of children and adolescents in Germany have at some point in their lives evidently been diagnosed with an atopic disease. More than one in six children (16.1%) remain currently affected by one of these three diseases. This prevalence has not changed since the KiGGS baseline study (16.1% vs. 15.6%) and in absolute figures affects 2.1 million children and adolescents in Germany.

#### Allergic contact dermatitis

Whereas in the KiGGS baseline study the data on allergic contact dermatitis was self-reported, KiGGS Wave 2 asked whether an allergic contact dermatitis had ever been medically diagnosed and whether the disease had appeared during the last 12 months. As expected the lifetime (2.8%) and 12-month (1.2%) prevalences are lower. For girls, the lifetime prevalences tend to be higher than for boys (3.2% vs. 2.4%; Table 1). These differences are statistically significantly higher for 7- to 10-year-old and 14- to 17-year-old girls compared to boys of the same age. There are, however, no differences in the 12-month prevalence of allergic contact dermatitis between gender and age groups (Table 2). In absolute figures 150,000 girls and boys are currently affected by allergic contact dermatitis in Germany.

#### Allergic sensitisation to inhalant allergens

37.1% of 3- to 17-year-olds are sensitised to SX1, a mix of the eight frequent inhalant allergens timothy grass, rye, birch, mugwort, cat and dog epithelial, house dust mites and the fungus cladosporium herbarum. The proportion of SX1 sensitised boys (42.6%) is higher than girls (31.3%). For both genders, the prevalence of SX1 sensitisation increases continuously with age. More than half of all boys aged between 14 and 17 (55.7%) present a potential for allergy (Table 2).

The sensitisation to frequent inhalant allergens is not dependent on the SES of families of children and adolescents. The region (East or West) also has no influence on SX1 sensitisation.

**Figure 4:**
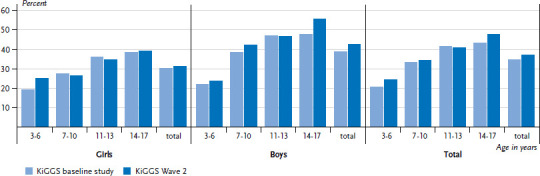
Trends in the prevalence of allergic sensitisation to the SX1 mix of allergens between the KiGGS baseline study and KiGGS Wave 2 according to gender and age (KiGGS baseline study n=6,348 girls, n=6,687 boys; KiGGS Wave 2 n=1,499 girls, n=1,463 boys) Source: KiGGS baseline study (2003-2006), KiGGS Wave 2 (2014-2017)

Compared to the KiGGS baseline study, the prevalence of SX1 sensitisation among children and adolescents has increased slightly although not statistically significantly (KiGGS Wave 2 37.1%, KiGGS baseline study 34.7%). The gender and age specific development of trends shown in Figure 4 reveals that SX1 sensitisation has increased most strongly among 14- to-17-year-old boys (55.7% vs. 47.7%; p=0.03).

## 4. Discussion

Following the well-known trend of a clear increase during the second half of the 20th century, allergic diseases have become prominent diseases relevant to public health. Population measures as an adequate response that strives to increase knowledge about allergic diseases, promote prevention, diagnosis and therapy requires a continuous flow of generated key epidemiologic figures on the prevalence of allergies. Since the turn of the century, population-based health monitoring at the Robert Koch Institute has decisively contributed to generating data on allergic diseases in Germany.

Most recent data from KiGGS Wave 2 on the current prevalence (12-month prevalence) of the most common allergic diseases such as hay fever (8.8%), atopic dermatitis (7.0%) and bronchial asthma (3.5%) among children and adolescents reveal that no significant changes have occurred relative to the KiGGS baseline study, which indicates a stabilisation of allergy prevalence at a high level. To a certain degree, gender and age-specific developments are however noteworthy, for example the greater prevalence of asthma among 7- to 13-year-old boys compared to the prevalence found among boys of the same age around ten years previously. Current results by no means indicate a change of trend. Today around one in six children (16.1%) are affected by at least one of these three diseases. In absolute figures, this translates into over 2.1 million children and adolescents in Germany.

Figures for allergic sensitisation based on measuring IgE antibodies in the blood allows conclusions on the potential for allergies among children and adolescents in Germany to be drawn. In particular sensitisation to inhalant allergens plays a role in the development of hay fever and asthma. Although allergic sensitisation in itself is not a disease, it is nonetheless the basis for developing symptoms. The prevalence of sensitisation to a mix of eight frequent inhalant allergens shows potential for allergies in one in two adolescents. Similar to the development of disease prevalence, the prevalence of SX1 sensitisation over the course of the last ten years has remained stable at a high level. Unlike in girls, the trend for boys has seen a slight increase.

The degree to which individuals maintain allergic sensitisations over their life course, newly develop or lose sensitisations is an important research question that can be analysed based on the KiGGS cohort data. Initial results indicate that new sensitisations to SX1 allergens occur significantly more often than remissions and that once acquired, sensitisations usually remain [15]. As sensitisations do not seem to disappear to any significant degree with age, providing healthcare to people affected by allergies will most likely become an increasingly important field of action for the health care system of years and decades to come, even if prevalences of those of similar age stop increasing over time.

The prevalences estimated currently for Germany among children and adolescents are reflected in the results of regional examinations such as the school entry examinations in Bavaria in 2012/2013 [16]. Comparisons of prevalences with studies from other European and non-European countries are only possible to a very limited degree due to the use of different survey instruments in different study populations based on diverging case definitions as well as different examination periods. The dimension of prevalences in Germany, however, does correlate quite well with those reported by other European countries [17-24]. To gain meaningful and representative results on prevalence and trends of allergic diseases in Germany, regular surveying in the context of health monitoring that is based on a comparable methodology will be required. Going beyond disease prevalence, future monitoring studies will survey the prevalence of symptoms. Not only will this increase the disease specificity of data, it will also lead to a better comparability with other symptoms-related surveys such as, for example, the ISAAC study, the largest international study so far on the spread of asthma and allergies among children and adolescents. A regular comparison of reliable data on the health, health behaviour and care as well as socioeconomic variables at the European level, the European Health Interview Survey (EHIS) has been developed. EHIS data is collected based on uniform standards and applies and further develops harmonised surveying tools across various countries [25].

In addition to increasing disease specificity by improving questions, it is currently being considered whether the format in which data is regularly collected in KiGGS studies, which is based on a standardised collection of data on diseases through reporting, should or could be validated by medical diagnoses through corresponding data sources for example billing data.

A principal strength of the available cross-sectional analyses is that the observed results can be generalised to reflect the German population from sampling, implementation and weighting. As in all surveys, bias due to selective non-participation is possible. As far as trend analyses are concerned, possible effects from changing the survey format from the KiGGS baseline study to KiGGS Wave 2 have to be considered. Whereas in the KiGGS baseline study, a personal interview with a physician took place during the examination period, participants in KiGGS Wave 2 were either personally interviewed or received a disease questionnaire by post to fill in. The analyses of responses of both sample groups (interview sample/interview and examination sample) revealed no significant deviations in the prevalence of allergic diseases meaning that a joint analysis of both groups became possible.

Current developments of the spread of allergic diseases among children and adolescents in Germany clearly show the continuously high number of cases. For patients, their families and the interested public, the Helmholtz Zentrum München has been developing its allergy information service in cooperation with the Federal Ministry of Health since 2017 to provide, via the internet, readily understandable information that is up to date and scientifically secured from the fields of allergy research and allergology [26]. From an academic perspective, the current guideline on specific immunotherapy only deals with the existing causal therapy for allergies. On its website, the German Society for Allergology and Clinical Immunology (DGAKI) updates the guideline every six months with updated information on specific medicines [27]. In general, an early diagnosis and adequate care for allergic diseases is not only important for those suffering from allergies but also an economically relevant issue.

## Key statements

- The prevalence of hay fever, asthma and atopic dermatitis among children and adolescents has remained stable in Germany between the KiGGS baseline study (2003-2006) and KiGGS Wave 2 (2014-2017).
- 16% of children and adolescents currently suffer from hay fever, asthma and/or atopic dermatitis. This corresponds to more than 2.1 million children and adolescents in Germany.
- One in two adolescents in Germany is allergically sensitised.
- The high number of people affected throws up a great deal of future challenges to the health system.

## Figures and Tables

**Table 1:** Life time prevalences of allergic diseases according to gender, age, socioeconomic status and region of residence Source: KiGGS Wave 2 (2014-2017)

	Asthma (n=7,400 girls, n=7,317 boys)		Hay fever (n=7,437 girls, n=7,368 boys)		Atopic dermatitis (n=7,386 girls, n=7,343 boys)		Allerg. contact dermatitis (n=7,341 girls, n=7,254 boys)	
	%	(95 % Cl)	%	(95 % Cl)	%	(95 % Cl)	%	(95 % Cl)
**Total** **(girls and boys)**	**6.0**	**(5.5-6.6)**	**11.0**	**(10.3-11.8)**	**12.8**	**(12.1-13.6)**	**2.8**	**(2.5-3.2)**
Girls	4.5	(3.9-5.2)	8.9	(8.0-9.8)	12.6	(11.6-13.7)	3.2	(2.7-3.8)
Boys	7.5	(6.6-8.4)	13.0	(11.9-14.2)	13.1	(12.0-14.2)	2.4	(2.0-2.9)
**Region of residence**								
East	5.1	(4.2-6.1)	9.8	(8.8-11.0)	15.7	(14.4-17.2)	2.1	(1.7-2.8)
West	6.2	(5.6-7.0)	11.3	(10.4-12.2)	12.2	(11.3-13.1)	2.9	(2.5-3.4)
**Socioeconomic status**								
Low	6.5	(5.1-8.3)	10.6	(8.9-12.6)	11.1	(9.3-13.3)	2.5	(1.7-3.7)
Medium	6.5	(5.8-7.4)	11.6	(10.7-12.6)	12.7	(11.8-13.7)	3.0	(2.5-3.6)
High	4.2	(3.6-4.8)	9.5	(8.5-10.6)	14.9	(13.5-16.4)	2.6	(2.0-3.3)
**Age group** **(girls and boys)**								
0-2 Years	1.1	(0.5-2.5)	3.0	(1.9-4.7)	11.2	(9.2-13.5)	2.1	(1.2-3.7)
3-6 Years	3.6	(2.8-4.5)	4.5	(3.7-5.6)	12.4	(11.0-13.0)	1.9	(1.4-2.7)
7-10 Years	6.3	(5.1-7.6)	10.8	(9.4-12.3)	13.6	(12.1-15.1)	3.0	(2.4-3.9)
11-13 Years	8.4	(6.9-10.1)	15.6	(13.7-17.6)	12.7	(11.2-14.4)	3.4	(2.6-4.3)
14-17 Years	9.5	(8.3-10.8)	19.1	(17.3-21.0)	13.7	(12.5-15.0)	3.4	(2.7-4.2)
**Girls**								
0-2 Years	0.9	(0.2-3.4)	3.3	(1.8-6.0)	10.1	(7.7-13.1)	1.4	(0.5-3.6)
3-6 Years	3.0	(2.0-4.5)	3.0	(2.1-4.1)	11.8	(9.9-14.0)	1.5	(0.8-2.6)
7-10 Years	4.4	(3.1-6.3)	7.8	(6.2-9.7)	13.6	(11.4-16.0)	4.2	(3.0-5.7)
11-13 Years	5.0	(3.8-6.7)	12.3	(10.1-15.0)	12.5	(10.6-14.8)	3.4	(2.4-4.7)
14-17 Years	7.8	(6.4-9.5)	16.6	(14.2-19.3)	14.0	(12.2-16.1)	4.8	(3.7-6.2)
**Boys**								
0-2 Years	1.4	(0.5-3.6)	2.8	(1.4-5.5)	12.2	(9.0-16.3)	2.8	(1.3-5.6)
3-6 Years	4.1	(3.0-5.5)	6.1	(4.6-7.9)	12.9	(10.9-15.3)	2.4	(1.5-3.7)
7-10 Years	8.0	(6.5-9.9)	13.6	(11.4-16.0)	13.6	(11.6-15.8)	1.9	(1.3-2.8)
11-13 Years	11.4	(9.1-14.3)	18.6	(15.7-22.0)	12.9	(10.8-15.2)	3.3	(2.2-4.9)
14-17 Years	11.0	(9.3-13.0)	21.4	(18.8-24.2)	13.4	(11.5-15.6)	2.1	(1.4-3.0)

CI = Confidence interval

**Table 2:** 12-month prevalence of allergic diseases and sensitisation to inhalant allergens (SX1 test) according to gender, age, socioeconomic status and region of residence Source: KiGGS Wave 2 (2014-2017)

	Asthma (n=7,402 girls, n=7,317 boys)		Hay fever (n=7,431 girls, n=7,367 boys)		Atopic dermatitis (n=7,381 girls, n=7,341 boys)		Allerg. contact dermatitis (n=7,343 girls, n=7,266 boys)		Sensitisation* (n=1,499 girls, n=1,463 boys)	
	%	(95 %-KI)	%	(95 %-KI)	%	(95 %-KI)	%	(95 %-KI)	%	(95 %-KI)
**Total** **(girls and boys)**	**3.5**	**(3.1-4.0)**	**8.8**	**(8.2-9.5)**	**7.0**	**(6.4-7.6)**	**1.2**	**(1.0-1.5)**	**37.1**	**(34.6-39.7)**
Girls	2.6	(2.2-3.2)	7.2	(6.4-8.1)	7.4	(6.6-8.3)	1.2	(1.0-1.5)	31.3	(28.3-34.3)
Boys	4.4	(3.7-5.2)	10.4	(9.4-11.4)	6.6	(5.8-7.4)	1.2	(0.9-1.6)	42.6	(39.0-46.4)
**Region of residence**										
East	3.4	(2.8-4.2)	8.0	(7.2-8.9)	9.4	(8.5-10.3)	1.1	(0.8-1.4)	37.0	(33.2-40.9)
West	3.6	(3.1-4.1)	9.0	(8.3-9.8)	6.4	(5.8-7.1)	1.3	(1.0-1.5)	37.1	(34.2-40.2)
**Socioeconomic status**										
Low	3.5	(2.5-4.9)	8.4	(6.8-10.2)	5.8	(4.4-7.5)	1.2	(0.7-2.1)	34.4	(28.8-40.5)
Medium	4.1	(3.5-4.7)	9.4	(8.6-10.2)	7.0	(3.3-7.8)	1.3	(1.0-1.6)	37.6	(34.3-41.0)
High	2.2	(1.7-2.7)	7.6	(6.7-8.6)	8.1	(7.1-9.2)	1.1	(0.7-1.6)	37.6	(33.1-42.4)
**Age group** **(girls and boys)**										
0-2 Years	1.1	(0.5-2.5)	2.8	(1.7-4.4)	10.0	(8.0-12.3)	1.6	(0.8-2.8)		-
3-6 Years	2.6	(2.0-3.4)	3.3	(2.6-4.3)	8.0	(6.9-9.2)	0.9	(0.6-1.4)	24.4	(19.6-29.9)
7-10 Years	4.3	(3.3-5.5)	8.7	(7.5-10.1)	7.0	(6.0-8.3)	1.2	(0.8-1.8)	34.4	(30.1-38.9)
11-13 Years	5.1	(4.0-6.5)	12.4	(10.6-14.4)	5.5	(4.6-6.5)	1.2	(0.8-1.8)	40.9	(36.4-45.6)
14-17 Years	4.2	(3.4-5.1)	15.3	(13.7-17.0)	5.1	(4.3-5.9)	1.3	(0.9-1.8)	47.7	(43.3-52.2)
**Girls**										
0-2 Years	0.9	(0.2-3.4)	3.2	(1.7-5.9)	8.8	(6.5-11.8)	0.8	(0.3-2.0)		-
3-6 Years	2.2	(1.5-3.3)	2.0	(1.3-3.0)	8.2	(6.6-10.2)	0.9	(0.5-1.7)	24.9	(18.9-31.9)
7-10 Years	2.8	(1.8-4.4)	6.0	(4.6-7.8)	8.3	(6.7-10.3)	1.4	(0.8-2.2)	26.2	(20.9-32.2)
11-13 Years	3.0	(2.0-4.5)	10.4	(8.3-13.0)	5.7	(4.4-7.4)	1.1	(0.6-2.0)	34.5	(28.6-41.0)
14-17 Years	3.7	(2.8-5.0)	13.3	(10.9-16.0)	6.0	(4.9-7.5)	1.8	(1.2-2.7)	39.0	(33.3-45.2)
**Boys**										
0-2 Years	1.4	(0.5-3.6)	2.4	(1.1-5.1)	11.0	(8.0-15.1)	2.3	(1.1-4.8)		–
3-6 Years	3.0	(2.1-4.1)	4.6	(3.4-6.2)	7.8	(6.4-9.3)	1.0	(0.5-1.8)	23.9	(17.8-31.4)
7-10 Years	5.7	(4.4-7.4)	11.3	(9.4-13.6)	5.8	(4.7-7.3)	1.1	(0.6-1.9)	42.3	(35.8-48.9)
11-13 Years	7.1	(5.2-9.5)	14.2	(11.5-17.4)	5.2	(4.1-6.7)	1.2	(0.6-2.4)	46.9	(40.0-54.0)
14-17 Years	4.6	(3.4-6.1)	17.2	(15.1-19.5)	4.2	(3.2-5.4)	0.8	(0.4-1.7)	55.7	(49.3-62.0)

CI = Confidence interval

* Age range 3-17 years
